# Use of interrupter technique in assessment of bronchial responsiveness in normal subjects

**DOI:** 10.1186/1471-2466-4-11

**Published:** 2004-11-12

**Authors:** Panagiotis Panagou, Ioannis Kottakis, Argyris Tzouvelekis, Stavros Anevlavis, Demosthenes Bouros

**Affiliations:** 1 Department of Pneumonology, Army General Hospital, Athens, Greece; 2 Department of Pneumonology, Medical School, University of Thrace, Alexandroupolis, Greece

## Abstract

**Background:**

A number of subjects, especially the very young and the elderly, are unable to cooperate and to perform forced expiratory manoeuvres in the evaluation of bronchial hyperresponsiveness (BHR). The objective of our study was to investigate the use of the interrupter technique as a method to measure the response to provocation and to compare it with the conventional PD_20 _FEV_1_.

**Methods:**

We studied 170 normal subjects, 100 male and 70 female (mean ± SD age, 38 ± 8.5 and 35 ± 7.5 years, respectively), non-smoking from healthy families. These subjects had no respiratory symptoms, rhinitis or atopic history. A dosimetric cumulative inhalation of methacholine was used and the response was measured by the dose which increases baseline end interruption resistance by 100% (PD_100_Rint, EI) as well as by percent dose response ratio (DRR).

**Results:**

BHR at a cut-off level of 0.8 mg methacholine exhibited 31 (18%) of the subjects (specificity 81.2%), 21 male and 10 female, while 3% showed a response in the asthmatic range. The method was reproducible and showed good correlation with PD_20_FEV_1 _(r = 0.76, p < 0.005), with relatively narrow limits of agreement at -1.39 μmol and 1.27 μmol methacholine, respectively, but the interrupter methodology proved more sensitive than FEV_1 _in terms of reactivity (DRR).

**Conclusions:**

Interrupter methodology is clinically useful and may be used to evaluate bronchial responsiveness in normal subjects and in situations when forced expirations cannot be performed.

## Background

It is known that assessment of bronchial responsiveness incorporating measurements of forced expiration can be problematic because of limited co-operation and fatigue and dizziness due to repeated forced expiratory manoeuvres. In addition, a deep inspiration, as it is required during an FEV_1 _(Forced Expiratory Volume in 1 sec) procedure, causes transient bronchodilatation particularly in normals during challenge with pharmaceutical substances, resulting in interpretation difficulties [1]. Determining bronchial reactivity using a technique which measures airways resistance is less influenced by inspiratory and expiratory efforts. Furthermore, it is more sensitive to small changes in bronchoconstriction [2] and hence more suitable for studies in normal subjects, in whom the response to bronchoconstrictors is, limited [3].

The interrupter method has been shown to be a simple and non-invasive technique of measuring airway mechanics in children or patients with limited co-operation [4]. It is also suitable for diagnostic purposes in the detection and exclusion of asthma [5] and in obtaining valid rhinomanometric measurements in various groups of patients [6].

An official statement by the ATS (American Thoracic Society) on methacholine provocation indicated that the interrupter method may be useful in testing patients who cannot perform acceptable spirometry manoeuvres but its use should be restricted to laboratories with expertise in their application and interpretation [7]. Furthermore, concerns have been raised about pressure equilibration during flow interruption [8] and when small increases in resistance are used as provocation thresholds, the repeatability of the method was found unacceptably low and unsuited for clinical and research purposes [9]. In addition, the studies performed so far with this technique were done on too small numbers of subjects to allow firm scientific conclusions.

We hypothesized that, since normals present with lower levels of airway obstruction during challenge, the interrupter technique in this case might be suitable and comparable with the reference PD_20_FEV_1 _method, and therefore clinically useful.

## Methods

### Subjects

The study was conducted in a tertiary referral centre for respiratory disease and 198 subjects were initially enrolled. All subjects were healthy with a negative history and physical examination, normal blood counts, chemistries, chest radiography and spirometry.

One individual from this sample reacted to the diluent control solution (0.6%), defined as a resistance difference of > 30% baseline [9] and was excluded together with non evaluable data from 27 subjects. The final data of 170 normals were finally included, consisting of 100 (59%) males and 70 (41%) females. Predicted values for spirometry were obtained according to the European Community Coal and Steel (ECCS) [10]. All participants were given detailed information of the purpose of the study, which was approved by the hospital ethics committee, and signed a consent form. They were asked to come in the next morning, avoiding all factors listed in the ATS guidelines [7] that might cause a false negative test.

### Methods

Routine spirometry was performed according to standardized guidelines [11]. Interrupter resistance was measured at end interruption (Rint, EI) using the technique by Phagoo et al.[12], who showed that the Rint, EI reflected changes in lung mechanics more sensitively, than interrupter resistance measured at mid or begining of interruption. The Rint, EI is calculated from the airway opening pressure (time function) signal airway opening pressure Pao(t) as follows: based on the assumption that, during a brief (100 ms) airflow interruption there is equilibration between alveolar pressure (PA) and Pao, the Rint, EI is obtained by dividing the change in pressure by the immediately preceding flow. In this study we used the alternative method of opening the interrupter [13], which calculates Rint, EI from the Pao signal using a calibration resistance.

The airflow interruptions were performed using the Bronchoscreen system (Jaeger, Würzburg, Germany) [8], a computerized apparatus with a combined nebulizer-shutter head, which allows the changes in resistance of the respiratory system Rint, EI) to be measured with each breath. During quiet breathing, the opening interrupter Rint, EI was calculated. The seated subject (with noseclip in place and the cheeks partly supported by a rubber mouthpiece) breathed in a relaxed manner (in order to avoid glottic artifacts) ambient air to get accustomed to the apparatus. The shutter closed within 15 ms. The time of complete airflow interruption was 100 ms. It was triggered 150 ms after the onset of expiration. The dead space of the apparatus was 0.35 L. The pressure transducer (Honeywell 142 PC 01G; Chesham, Bucks, UK) was connected via a side port directly to the mouthpiece at a distance of 18 cm from the airway opening. Rint, EI was calculated by the formula: Rint, EI = (PA/Pm) × Rref, where PA is the end interruption mouth pressure, Pm the pressure generated during free flow and Rref is a fixed serial resistance. The triggering volume was determined by integrating the signal from a low resistance Lilly Pneumotach, which had a linearity of ± 2% at a flow below 12 L/s. Before each challenge the interrupter was calibrated. A vent produced an airflow of 105 L/min, which was led through the shutter and a calibrating resistance (0.10 kPa/L/s) and the determined Rint,EI had to be within ± 10% of the reference resistance. The above method has been found valid in the presence of mild to moderate bronchoconstriction [14], conditions that are normally met during bronchial challenge.

Bronchial responsiveness was measured by a rapid methacholine provocation dosimetric test, as previously described by our group with the same apparatus but using histamine instead [15]. Briefly, 1% methacholine in saline (Lofarma, Italy) was inhaled in doubling doses starting from 200 μg, until FEV_1 _had fallen = 20% compared with FEV_1 _after an initial saline inhalation. The bronchial aerosol provocation system (APS Jaeger, Wurzburg, Germany) was used in this procedure. The nebulizer was calibrated to draw 5 μL of solution per automatic actuation lasting 0.6 seconds. The 100 μL of aerosol bolus had a mass median aerodynamic diameter of 1.9 μm with 80% of the droplets being less than 5.5 μm at a set pressure of 1.6 bar (22.8 psi). The subjects inhaled methacholine by slow inspiratory capacity manoeuvres guided by the green colour of light emission diodes (so that inspiratory flow was <0.5l/s) and the response was assessed 1 min after each inhaled dose.

Data was assessed by using two different estimates: 1) provocation dose which increases Rint, EI by 100% (PD_100_Rint, EI), calculated by interpolation from the last two points of the cumulative semilogarithmic dose-response diagram, and 2) the percent slope (dose-response ratio-DRR) of a line extending from the origin to the last point of the curve (DRR) [16]. Plateau response was defined as difference in Rint, EI <40% after the delivery of three consecutive doubling doses and/or DRR<40% after a total cumulative dose of 4 mg or a PD_100 _Rint, EI >4 mg. The DRR data were analyzed from the whole sample. The 10 day reproducibility of the PD_100_Rint, EI was investigated by randomly asking 39 subjects to come again after one week for a second examination. During the second visit we compared Rint, EI with FEV_1 _as measurements of response to provocation, the latter determined 30 s after the assessment of Rint, EI [9,17]. At least two technically correct forced expiratory manoeuvres with an FEV_1 _variation within ± 5% were received and the highest value was used for calculating the dose producing a 20% fall in FEV_1 _(PD_20_FEV_1_).

### Statistical analysis

Regression analysis and correlation, Shapiro-Wilk test for normality and the non-parametric Mann-Whitney-U-/Wilcoxon Rank Sum test with normal approximation and the x^2 ^test, were used for statistical analysis. The relative duplicate error was used to assess test-retest reproducibility of the PD_100_Rint, EI (assuming a normal distribution), defined as a standard deviation of the differences divided by the v2 after log transformation (approximates coefficient of variation)[18]. Agreement between PD100Rint, EI and PD_20_FEV_1 _was defined and calculated according to Bland and Altman [19]. Normal bronchial responsiveness was defined at a cut-off level of > 0.8 mg methacholine [20], while negative non-response reactions were those > 2.0 mg (8.8 μmoL)[21].

## Results

**Figure 1:**
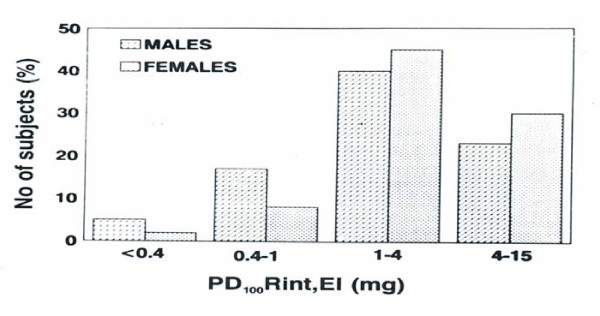
Analysis of the distribution of PD_100_Rint, EI (threshold dose) in males and females. Values >4 mg are derived by extrapolation.

Subjects' anthropometric data and baseline spirometry are shown in Table 1. Mean values of vital capacity (VC), FEV_1 _and maximal expiratory flow when 50% of the forced vital capacity (FVC) remains to be exhaled (Vmax_50_), were higher in males by 16.3%, 14, 7% and 2.5% than in females. While Rint, EI was higher in females, possibly reflecting smaller airway size, but these differences were not statistically significant. The distribution of PD_100_Rint, EI is shown in Figure 1.

**Table 1:** Characteristics of the study population stratified by gender.

**Variables**	**Men**	**Women**
	**n = 100**	**n = 70**

**Age, mean (range) yr**	38 (18–60)	35 (18–55)
**Height, mean (cm), SEM**	174 (0.78)	160 (0.71)
**Weight, mean (kg), SEM**	79 (0.9)	63 (0.9)
**Rint, EI, mean (kPa/l/s), SEM**	0.24 (0.069)	0.29 (0.074)
**VCin, mean (%pred), (range)**	111.5 (83–144)	95.2 (76–129)
**FEV_1_, mean(%pred), (range)**	107.9 (75–125)	93.2 (78–110)
**FEV_1 _% mean (range)**	83 (77–92)	82 (75–90)
**Vmax_50_, mean (%pred), (range)**	83.5 (70–155)	81 (65–145)

***Abbreviations***: VC in: inspiratory vital capacity, FEV_1_: forced expiratory volume in 1 sec, FEV_1_%: ratio of forced expiratory volume in 1 sec to forced vital capacity, Vmax_50_: maximum flow at 50% of forced vital capacity, Rint, EI:Interrupter Resistance at End Interruption

Twenty one males and ten females (18%) of our subjects exhibited bronchial hyperesponsiveness. These values were normally distributed (W = 0.93, p = 0.12), with no gender related difference (x^2 ^= 1.48, p = 0.22, odds = 1.79). Furthermore, 5 of these 31 subjects (3 men and 2 women, 3% of total) were found to show moderate bronchial hyperesponsiveness (PD_100_Rint, EI < 0.4 mg or < 1.66 μmol methacholine), as frequently found in current symptomatic asthmatics [21]. No correlation was found of PD_100_Rint, EI to baseline post-saline Rint, EI and the respective DRR. Subjects with negative reactions (> 8.8 μmol) showed DRRs that were ten times lesser compared to those with BHR (mean ± SD = 67.52 ± 10.66 vs 690 ± 390, p < 0.001). Plateau response was exhibited by 66 (38%) of the subjects, (36 males) without gender related statistical difference (x^2 ^= 0.81, p= 0.36). They had DRRs that were 2.5 times smaller compared to the subjects with normal but measurable reactions (30.1 ± 9.8 vs. 75.0 ± 49.9, p = 0.024).

**Figure 2:**
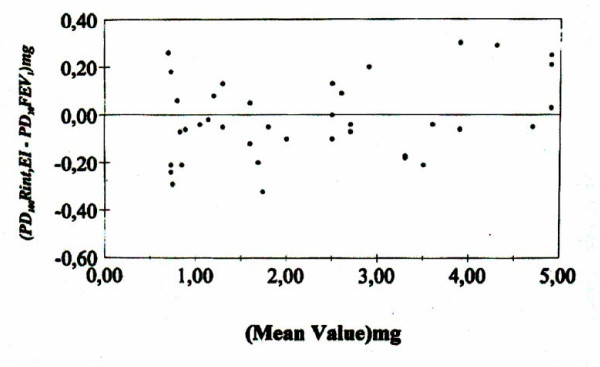
Bland and Altman plot of the differences between two methods against their mean value. The limits of agreement ( -2s and  +2s) are -0.334 mg (-1.39 μmol) and 0.306 mg (1.27 μmol) respectively. The 95% confidence intervals are -0.364 to -0.303 mg and 0.275 to 0.336 mg, respectively.

PD100Rint, EI was found reproducible with a duplicate error of 8.3% or 0.65 doubling doses (within 140 μg). A close correlation was found between PD_100_Rint, EI and PD_20_FEV_1 _(r = 0.76, 95% CI 0.53-0.88) with relatively narrow limits of agreement (Figure 2.) Stratification of data according to BHR status is shown in Table 2. The interrupter method showed DRRs that were more reactive in comparison to the respective DRRs of FEV_1_(approximately seven-fold).

**Table 2:** Comparison of the methods described in the text in terms of threshold dose (sensitivity) and dose-response ratio (reactivity), stratified according to BHR status. NS: p value not statistically significance between the two methods. The greater reactivity of the interrupter method is shown.

**Methods (× ± SD)**			
**Subjects showing BHR**			
PD_100_Rint, EI (mgs)	0.57 ± 0.20	Dose-response ratio (%/mg)	690 ± 390
PD_20_FEV_1 _(mgs)	0.72 ± 0.66	Dose-response ratio (%mg)	98 ± 90
	NS		P < 0.05
**Subjects with normal measurable reactions (> 0.8 mgs)**			
PD_100_Rint, EI (mgs)	3.42 ± 3.10	Dose-response ratio (%/mg)	74.86 ± 49.87
PD_20_FEV_1 _(mgs)	3.13 ± 2.65	Dose-response ratio (%/mg)	20 ± 3.82
	NS		P < 0.05

***Abbreviations***: BHR :Bronchial Hyperresponsiveness, Rint, EI:Interrupter Resistance at End Interruption, PD_20_FEV_1_:Dose Producing a 20% fall in FEV_1_.

## Discussion

In this study we have shown that the interrupter technique, and specifically PD_100_Rint, EI, is comparable to the conventional PD_20_FEV_1 _method for evaluation of BHR in a large sample of normal subjects. We have also found that this technique has a specificity of 81.2% for normal subjects and its dose response ratio is 7-fold more sensitive than the conventional FEV_1 _method. It is known that specific airway conductance is four times more sensitive than FEV_1 _as a measure of response to provocation but the use of a body plethysmograph makes assessment of bronchial challenge expensive and time consuming. The present methodology is particularly useful in children and in the elderly, since it is non-invasive, sensitive to changes in airway calibre and requires no subject co-operation. The opening interrupter technique offers the additional advantage of simplicity and ease of application, being particularly useful in subjects unable to perform forced manoeuvres.

The Rint, EI was measured during expiration above forced residual capacity (FRC), because resistance hardly changes above this level and since subjects performed relaxed tidal flow maoeuvres, measurements were not affected by variations in breathing. Furthermore, we did not correct Rint, EI by lung volumes because the variability formed in the FRC can reduce the benefit of standardization of Rint, EI and the correlation between respiratory resistance and FRC is not significant over the limited FRC range of healthy subjects [21]. Problems in repeatability have been reported when one uses provocative concentration causing a 30% increase in Rint, EI (PC_30_Rint, EI), so we assessed the PD_100_Rint, EI threshold, which is above the 95 % confidence interval at one tail direction (1.96SD) observed in our normal sample at baseline (Table 1). Furthermore, although a correlation of the PC_40_Rint, EI with the classical provocative concentration causing a 20% fall in FEV_1 _(PC_20_FEV_1_) has been reported (17), data on agreement are presented for the first time in this study.

If a cut-off value is set at 0,4 mg PD_100_Rint, EI methacholine, which defines severe and moderate hyperresponsiveness compatible with asthma [22] then 3% of the studied normal population was found to be in this area. This is similar to the percentage found by Malo et al. [23] working with PC_20_FEV_1 as _well as to 2.5%, which represents the proportion of subjects beyond the 2 SD of the mean on one side of a normal distribution. If the PD_100_Rint, EI threshold is set at 0,8 mg methacholine [18], which includes mild BHR, then 18% of the total subjects studied had some degree of hypersensitivity. Contradictory results have been previously reported regarding the clinical significance of asymptomatic BHR. Some studies, using even stricter definitions of BHR, showed that asymptomatic BHR is of no significance [24]. In contrast other studies have reported an increased rate of decline of lung function in an asymptomatic population with BHR [25]. A plateau with a low maximal response was exhibited by 38% of the subjects, thus representing the least reactive part of the sample. Seppala et al.[26], using a method incorporating a deep inhalation (FEV_1_) showed that 50% of normals had no calculable PC_20_FEV_1_.

In this study, by direct comparison of two methods, one involving a deep inhalation, the relative effects of maximal expiratory manoeuvres on airway calibre can be indirectly assessed. The data in Table 2 show that while threshold doses between the two methods are essentially similar and not statistically significant, there is a significant difference in the DRRs between the two methods, being more pronounced in normal subjects showing BHR.

Recently, Sundblad et al [27] reported a significant correlation between dose response slopes of FEV_1 _and airway conductance in a large sample of subjects but not all were normals. It is known that in bronchial challenge the dose-response curve is expressed mainly by the threshold dose indicating hyperresponsiveness and the rate and magnitude of the response (hyperreactivity, DRR). The less reactive DRRs of the FEV_1 _method lend support to the perturbed actomyosin equilibrium hypothesis recently described, in that with stretching there is a decrease in myosin duty cycle and the magnitude of the contractile response becomes functionally disengaged from the level of the contractile stimulus [28]. Furthemore, since there is indirect evidence of a lack of airway inflammation or remodelling that could prevent smooth muscle from stretching, our data are in agreement with those of Kolnaar et al. [24]. The fact that airway elastic recoil decreases (increase in hysteresis) when smooth muscle is contracted [1], explains the greater difference in DRRs exhibited by subjects showing BHR (the prevailing distending force of the lung allows the airway to dilate more after deep inhalation).

No correlation was found in this study between BHR and baseline airway calibre, although Malo et al.[23] found a weak correlation by using a more sensitive parameter i.e. the PC_6 _FEV_1_. The limits of agreement between PD_100_Rint, EI and the classical PD_20_FEV_1 _were found relatively small at -1.39 μmol and 1.27 μmol respectively. This implies that this method may be used as an alternative to FEV_1 _during provocation, as it is simple and easy to perform and requires no patient co-operation.

Gender differences in BHR were explored because of the smaller lung size in women. Our data are in agreement with recent studies [29] that in non smoking women, lung size has no effect on bronchial sensitivity. Since RintL was found more sensitive than RintEI, a study comparing the two methods with the classical method would be interesting [30].

## Conclusions

In summary, the interrupter technique as an index of response to provocation has been shown to be useful to assess bronchial responsiveness in normal subjects, when maximal efforts cannot be performed. We recommend threshold doses of 100% baseline, because they show reliable agreement with the classical PD20FEV1 method.

## List of abbreviations

ATS:American Thoracic Society, BHR:Bronchial Hyperresponsiveness, DRR:Dose Response Ratio, FEV_1:_Forced Expiratory Volume in 1 sec, FVC:Forced Vital Capacity, FRC:Functional Residual Capacity, PA:Alveolar Pressure, Pao: airway opening Pressure, PC_20_FEV_1_:Provocative Concentration causing a 20% fall in FEV_1_, PC_30_Rint, EI:Provocative Concentration causing a 30% increase in Rint, EI, PD_100_Rint, EI: Provocation dose which increases Rint, EI by 100%, PD_20_FEV_1_:Provocation Dose producing a 20% fall in FEV_1_, Rint, EI:Interrupter Resistance at End Interruption, VC:Vital Capacity,

## Competing interests

The authors declare that they have no competing interests.

## Authors' contributions

PP, IK, AT, SA, DB were involved with the study conception. PP, AT, SA performed the interrupter technique and collected the data. PP did the statistical analysis. PP, AT, DB prepared the manuscript. All authors read and approved the final manuscript.

## Pre-publication history

The pre-publication history for this paper can be accessed here:


